# Simvastatin Reduces NETosis to Attenuate Severe Asthma by Inhibiting PAD4 Expression

**DOI:** 10.1155/2023/1493684

**Published:** 2023-02-02

**Authors:** Yun-Rong Chen, Xu-Dong Xiang, Fei Sun, Bo-Wen Xiao, Mu-Yun Yan, Biao Peng, Da Liu

**Affiliations:** ^1^Department of Pulmonary and Critical Care Medicine, Hunan Provincial People's Hospital (The First Affiliated Hospital of Hunan Normal University), Changsha, Hunan 410016, China; ^2^Department of Pulmonary and Critical Care Medicine, The Second Xiangya Hospital, Central South University, Changsha, Hunan 410011, China; ^3^The Center for Biomedical Research, Department of Respiratory and Critical Care Medicine, NHC Key Laboratory of Respiratory Diseases, Tongji Hospital, Tongji Medical College, Huazhong University of Science and Technology, Wuhan, Hubei 430030, China; ^4^Department of Interventional Vascular Department, The Affiliated Changsha Central Hospital, Hengyang Medical School, University of South China, Changsha, Hunan 410004, China; ^5^Department of Pulmonary and Critical Care Medicine, The Affiliated Changsha Central Hospital, Hengyang Medical School, University of South China, Changsha, Hunan 410004, China

## Abstract

**Objective:**

Patients with severe asthma respond poorly to corticosteroids, and their care accounts for more than 60% of the total costs attributed to asthma. Neutrophils form neutrophil extracellular traps (NETs), which play a crucial role in severe asthma. Statins have shown anti-inflammatory effects by reducing NETosis. In this study, we investigate if simvastatin can attenuate severe asthma by reducing NETosis and the underlying mechanism.

**Methods:**

Mice were concomitantly sensitized with ovalbumin (OVA), house dust mite (HDM), and lipopolysaccharide (LPS) during sensitization to establish a mouse model of severe asthma with neutrophil predominant inflammation (OVA+LPS mice) and treated with or without simvastatin. In inflammatory response, proportions of Th2, Th17, and Treg cells in lung tissue were detected by flow cytometry, and the levels of cytokines, dsDNA, and MPO-DNA in bronchoalveolar lavage fluid (BALF) were analyzed by ELISA. Citrullinated histone H3 (CitH3) and peptidyl arginine deiminase 4 (PAD4) in lung tissue were determined by Western blot and immunofluorescence imaging. PAD4 mRNA was determined by quantitative PCR (qPCR). HL-60 cells were differentiated into neutrophil-like cells by 1.25% DMSO. The neutrophil-like cells were treated with or without LPS, and simvastatin was then stimulated with PMA. CitH3 and PAD4 expressions were determined.

**Results:**

Sensitization with OVA, HDM, and LPS resulted in neutrophilic inflammation and the formation of NETs in the lungs. Simvastatin treatment reduced the inflammation score, cytokine levels, total cells, and neutrophil counts in the BALF and reduced proportions of Th2 and Th17 but increased Treg cells in lungs of OVA+LPS mice. Simvastatin-treated OVA+LPS mice show reduced NET formation in BALF and lung tissue compared to control mice. Adoptive transfer of neutrophils was sufficient to restore NETosis and neutrophilic inflammation in simvastatin-treated OVA+LPS mice. Simvastatin reduced PAD4 mRNA and protein expression in lung tissues and neutrophils isolated from lungs of OVA+LPS mice and consequent NET formation. In vitro, simvastatin reduced LPS-induced PAD4 upregulation and NETosis in HL-60-differentiated neutrophil-like cells. Furthermore, PAD4-overexpressed lentiviral transduction was sufficient to restore PAD4 protein expression and NETosis in simvastatin-treated HL-60-differentiated neutrophil-like cells.

**Conclusions:**

Simvastatin reduces Th17-mediated neutrophilic inflammation and airway hyperreactivity by reducing PAD4 expression and inhibiting NETosis in a mouse model of severe asthma. Severe asthmatic patients with high levels of circulating NETs or sputum NETs may show improved responses to statin treatment.

## 1. Background

Asthma is a common inflammatory disease of the airways with several clinical phenotypes and underlying endotypes [1–3]. About 10% of the population of adults with asthma are severe patients [1], but their medical care accounts for more than 60% of the total costs attributed to asthma [2]. Patients with severe asthma respond poorly to corticosteroids, which lead to frequent exacerbations, hospitalization, emergency department visits, and even severe complications such as respiratory failure and depressive disorder. The nature of severe asthma calls for the development of more effective therapies.

Severe asthma has been characterized by a Th17-mediated nontype 2 neutrophilic inflammation [4, 5]. In response to inflammatory stimulation, neutrophils form neutrophil extracellular traps (NETs), which contain decondensed DNA, citrullinated histone H3 (CitH3), and granule proteins such as myeloperoxidase (MPO) and neutrophil elastase. Studies have confirmed that NETs play a crucial role in allergic and severe asthma and that inhibition of NET formation (NETosis) is sufficient to decrease airway inflammation and airway responsiveness induced by methacholine (MCh) in both type 2 asthma and nontype 2 severe asthma [5–7]. Peptidyl arginine deiminase 4 (PAD4), a nuclear enzyme that drives the histone H3 citrullination and implicated in chromatin reorganization, is essential to NETosis [8].

Statins have showed anti-inflammatory and immunomodulatory effects on multiple levels in different diseases, including systemic lupus erythematosus, multiple sclerosis, rheumatoid arthritis, and asthma, from several independent studies [9]. Multiple large clinical studies in asthma have shown that statin use improved clinical outcomes, such as reduced oral corticosteroid use, hospitalization, emergency department visits, and age-related lung function decline [10–15]. Therefore, statins may become an affordable alternative therapy for the treatment of severe asthma [15]. However, several small randomized controlled trials focused on the efficacy of statins on the treatment of asthma have yielded conflicting results [16–18]. These findings raised questions about what is the reason of the conflicting results. This may attribute to the heterogeneity of asthma [15]. Hence, it is crucial for us to identify specific pathogenic mechanisms (endotypes) related to certain subpopulation of patients with severe asthma who responds well to statins.

A recent study by Sapey et al. showed that simvastatin reduced NETosis in patients with sepsis and improved their outcomes [19]. Another study by Al-Ghoul et al. found a decreased NET formation and a subsequent protective effect in a mouse model for thermal injury treated by simvastatin [20]. Therefore, we asked whether the treatment effect of statins on asthma is via inhibition of NETosis and, if so, what is the underlying mechanism for this inhibition.

Here, in a mouse model of severe asthma with Th17 cell-mediated neutrophilic inflammation, we found that simvastatin treatment ameliorated severe asthma by reducing NETosis in the lung of severe asthmatic mice. Furthermore, we determined the potential mechanisms underlying the impact of simvastatin on NETosis in asthmatic mice and HL-60-differentiated neutrophil-like cells.

## 2. Material and Methods

### 2.1. Mice

Female C57BL/6 mice (provided by Animal Center of Central South University, Changsha, China), aged 6-7 weeks, weighing 18–20 grams, were used for the asthma model. All mice were bred and housed in a SPF facility with a 12/12 h light/dark cycle, with diet freedom. The animal protocols were approved by the Institutional Animal Care and Use Committee of Changsha Central Hospital Affiliated to University of South China.

### 2.2. Mouse Model of Asthma

Mouse models of severe asthma and conventional asthma were established as described in our previous study with slight modifications [21, 22]. Female C57BL/6 mice were grouped according to a random number table, with six mice in each group. Mice in the severe asthma group (OVA+LPS group) were given an intraperitoneal sensitization injection with 100 *μ*g HDM+100 *μ*g OVA+15 *μ*g LPS on days 0, 1, and 2 and then challenged with an atomized OVA solution for 30 min before HDM intranasal excitation on days 14, 15, 18, and 19. For the control group, mice received saline only. Mice in the conventional asthma group (OVA group) were given a sensitization intraperitoneal injection with 25 *μ*g OVA on days 0 and 7 and then challenged with an atomized OVA solution excitation for 30 minutes on days 14, 15, 16, 17, 18, 19, and 20. All mice were sacrificed on day 21.

### 2.3. Treatment with Cl-amidine, DNase I, or Simvastatin In Vivo

Cl-amidine was injected i.p. (10 mg/kg in 200 *μ*l of 1% DMSO *v*/*v* in PBS) twice daily from day -1 to day 2 and from day 13 to day 20. Alternatively, DNase I (Sigma-Aldrich) was injected i.p. (1,000 IU in 200 *μ*l of PBS) once daily from day -1 to day 2 and from day 13 to day 20. Simvastatin was injected i.p. (40 mg/kg in 200 *μ*l of PBS) once daily from day -1 to day 2 and from day 13 to day 20.

### 2.4. Airway Hyperresponsiveness

Methacholine- (Mch-) induced airway resistance was measured on day 21 (24 h after the final challenge) by direct plethysmography (Buxco Electronics, RC System, USA). The procedures were the same as previously described [21]. Mice were anesthetized and mechanically ventilated. Then, mice were given aerosolized methacholine (0, 3, 10, 30, and 100 mg/ml) via the inhalation port for 10 s. The relative lung resistance for each dose of methacholine, normalized to the saline baseline, was calculated.

### 2.5. Bronchoalveolar Lavage Fluid (BALF) Cell Count

BALF collection was conducted as previously described [22]. In brief, the lung of the mice was flushed with 0.9 ml of cold PBS containing 2 mM EDTA and 2% fetal bovine serum (FBS). BALF was then centrifuged at 400 g at 4°C for 10 min. Cell pellets were resuspended and total BALF cell counts were calculated using a hemocytometer. The number of eosinophils and neutrophils was determined in 200 total BALF cells after H&E staining. The supernatant was collected for analysis of cytokine profile.

### 2.6. ELISA Measurement of Cytokines

The total leukocyte count and concentrations of IFN-*γ*, IL-1*β*, IL-4, and IL-17A in bronchoalveolar lavage fluid (BALF) were measured by murine cytokine-specific Quantikine ELISA kits (Proteintech, USA) in accordance with the manufacturers' instructions.

### 2.7. Measurement of Airway Inflammation Score and PAS Staining

After being fixed with 4% paraformaldehyde and embedded in paraffin, 4 *μ*m sections of lung tissues were stained with hematoxylin and eosin (H&E) (Beyotime, China) for standard histopathological examination. Each pathological section was observed under an optical microscope. The representative images for pathological analysis were utilized to evaluate the infiltration of inflammatory cells in the airways and perivascular and alveolar cells of mice. Paraffin-embedded lung sections were stained with periodic acid-Schiff (PAS) as previously described [21]. The extent of lung inflammation was scored for cellular infiltration around the airways: 0, no infiltrates; 1, few inflammatory cells; 2, one layer of inflammatory cells; 3, 2~4 layers of inflammatory cells; and 4, more than four layers of inflammatory cells. Mucus production was quantified as the percentage of PAS-stained goblet cells to total epithelial cells in randomly selected bronchi, as described [23].

### 2.8. Cell Isolation, Staining, and Flow Cytometry

To obtain single-lung-cell suspensions, the bronchial lungs of mice were collected, washed once with 5x antibiotic, washed twice with PBS, and cut into small pieces. Then, lungs were digested with collagenase1 (0.5 mg/ml Sigma) and 10 *μ*g/ml DNase I (Roche) in RPMI medium for 1 hour at 37°C in a water bath using a shaking incubator [21]. Blood was collected in an EDTA-containing tube (100 mM), and red blood cells were lysed with red blood cell lysis buffer (eBioscience, USA). Cells were filtered through a 70 *μ*M cell strainer to obtain single-cell suspensions.

The lung cells were then stained with surface marker FITC anti-CD4 cytokine antibody (Invitrogen, USA) followed by fixation and permeabilization with fixation and permeabilization buffer (eBioscience, USA) for 15 min. After washing with permeabilization buffer, the lung cells were stained with intracellular markers PerCP-Cy5.5-anti-IL17A and APC-anti-IL-4 cytokine antibodies (Invitrogen, USA) in the permeabilization buffer for 20 min. Flow cytometry was conducted and the data were analyzed using the FACS Calibur and FlowJo software.

### 2.9. Immunofluorescence Analysis

To determine the expression of PAD4 in neutrophils of lung tissues, we performed immunofluorescence staining on slides of paraffin-embedded lung tissues. The slides were incubated with primary antibodies overnight. Subsequently, the slides were cultured by Alexa Fluor 488 and Alexa Fluor 594 secondary antibodies (Invitrogen, USA) for 1 hour. Then, the slides were mounted by Vectashield (Vector Laboratories, USA) with DAPI. LSM 510 confocal microscope (Zeiss, Germany) was used for cell photography and counting.

To identify NETs from lung tissues, the slides were incubated with rabbit anti-mouse antibody directed against CitH3 (R&D Systems, 1 : 50 in blocking buffer) and with goat anti-mouse antibody directed against MPO (Abcam, 1 : 50 in blocking buffer) overnight. After washing samples with PBS, secondary antibodies, i.e., CoraLite488–conjugated Affinipure Goat Anti-Rabbit IgG (H+L) antibody (1 : 200 in blocking buffer) and CoraLite488–fluorescein (TRITC)-conjugated Affinipure Donkey Anti-Goat IgG (H+L) antibody (1 : 200 in blocking buffer) were added in blocking buffer (1 : 1,000) and the samples were incubated for 1 h. Then, the slides were mounted by Vectashield (Vector Laboratories, USA) with DAPI. LSM 510 confocal microscope (Zeiss, Germany) was used for cell photography and counting.

To identify NETs of neutrophils isolated from lungs, single lung cells were resuspended in HEPES-buffered RPMI medium, and neutrophils were isolated with a mouse neutrophil isolation kit (Solarbio, China). Next, neutrophils were fixed by 2% paraformaldehyde, permeabilized, blocked, and stained with rabbit anti-mouse antibody directed against CitH3 (R&D Systems, 1 : 50 in blocking buffer) and CoraLite488–conjugated Affinipure Goat Anti-Rabbit IgG (H+L) antibody (1 : 200 in blocking buffer). Then, the neutrophils were counterstained with DAPI and mounted by Vectashield (Vector Laboratories, USA). LSM 510 confocal microscope (Zeiss, Germany) was used for cell imaging and counting.

### 2.10. Adoptive Transfer of Neutrophils

For adoptive transfer experiments, mouse neutrophils were isolated from single-lung-cell suspensions using a mouse neutrophil isolation kit (Solarbio, China). Then, neutrophils were resuspended in calcium- and magnesium-free PBS at the concentration of 2 × 10^7^ cells/ml. 5 × 10^5^ cells were injected intratracheally to simvastatin-treated OVA+LPS mice in 50 *μ*l of PBS 30 min before OVA was injected from day –1 to day +2.

### 2.11. HL-60 Cell Culture

The human acute promyelocytic leukemia cell line HL-60 (Honorgene, China) was maintained in phenol red-free RPMI-1640 medium (sigma) supplemented with 10% FBS (Gibco, Australia) in 5% CO_2_ at 37°C. The lentiviruses were ordered from Honorgene (Changsha, China). HL-60 cells were infected with PAD4-overexpressing (PAD4^oe^) lentiviral particles or vector control (Vector). HL-60 cells were differentiated into neutrophil-like cells by culturing them in medium supplemented with 1.25% DMSO for 5 d. The level of HL-60 cell differentiation was evaluated by flow cytometry analysis of CD11b expression using a BD LSRFortessa Flow Cytometer and BD FACSDiva software. Differentiated neutrophil-like cells were incubated with simvastatin or PBS for 2 h and then incubated with LPS (100 ng/ml) or PBS for 2 h, followed by stimulation with PMA (100 nM) for 2 h. In some experiments, differentiated neutrophil-like cells transduced with PAD4 lentiviral particles or empty lentiviral particles were incubated with simvastatin for 2 h and then treated with LPS (100 ng/ml) for 2 h, followed by stimulation with PMA (100 nM) for 2 h.

### 2.12. Free Double-Stranded DNA (dsDNA) Measurement in BALF

DsDNA was measured in the cellular fraction of the BALF, which was obtained after double centrifugation and supernatant collection. Levels of dsDNA were determined with a mouse anti-double-stranded DNA antibody (IgG) ELISA Kit (Cusabio, China).

### 2.13. Western Blot

Lung tissues or HL-60-differentiated neutrophils were homogenized in RIPA buffer supplemented with a cocktail of protease inhibitors (Honorgene, China).

Determination of protein concentration was carried out by bicinchoninic acid protein assay kit (Pierce). After being subjected to sodium dodecyl sulfate-polyacrylamide gel electrophoresis, the protein was transferred to a nitrocellulose membrane. Total protein was detected by probing the membranes with indicated primary antibodies followed by incubation with an HRP-conjugated secondary antibody. The PAD4 and CitH3 antibodies were the same as above.

### 2.14. RT-qPCR

TRIzol® reagents (Invitrogen Life Technologies; Thermo Fisher Scientific, USA) were employed to extract total RNA. Subsequently, a PrimeScript™ RT reagent kit (Thermo Fisher Scientific) was utilized for reverse transcription. We conducted real-time RNA quantification on an ABI StepOne Plus Detection System (Applied Biosystems) using Power SYBR Green PCR Master Mix (Applied Biosystems). The primer sequences were designed in the laboratory and synthesized by Sangon Biotech Co., Ltd. (Shanghai, China). The primers were designed as follows: PAD4 forward, GATGCCTTTGGGAACCTGGA, and reverse, GCTGCTGGAGTAACCGCTAT, and actin forward, ACATCCGTAAAGACCTCTATGCC, and reverse, TACTCCTGCTTGCTGATCCAC.

### 2.15. Detection of Serum and BALF MPO-DNA Complex

Serum and BALF MPO-DNA complex were measured using a previously described capture ELISA method with slight modifications [24]. 96-well microtiter plates were coated with anti-MPO polyclonal antibody (1 : 1000, Invitrogen, USA) as the capturing antibody overnight at 4°C and washed 4 times with 0.05% PBS-Tween-20, then blocked with 1% BSA, and washed 3 times. Next, samples together with peroxidase-labelled anti-DNA monoclonal antibody were added (component No. 2 of the Cell Death Detection ELISA kit, Roche), and the wells were incubated at room temperature for 2 h and then washed with PBS 3 times. The peroxidase substrate (Roche) was added. After incubation at 37°C for 40 min, the optical density was measured at 405 nm using a microplate reader.

### 2.16. Statistical Analysis

The assumptions of normal distribution of residuals and homoscedasticity were verified, and data were presented as mean values ± standard deviation (SD). Statistical significance from different groups of mice was calculated by one-way ANOVA (>2 groups). Statistical analyses were performed with Prism 9 (GraphPad Software). We considered a *P* value lower than 0.05 as significant.

## 3. Results

### 3.1. Simvastatin Treatment Ameliorates Airway and Lung Inflammation in OVA+LPS Mice

**Figure 1:**
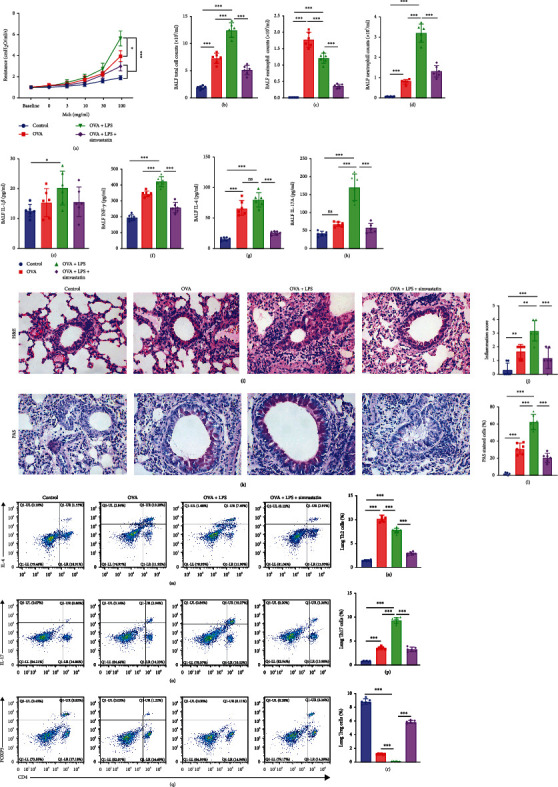
Simvastatin treatment ameliorates features of TH2-mediated and TH17-mediated asthma in mice. (a) Measurement of dynamic airway resistance. (b–d) Total cell counts, eosinophil counts, and neutrophil counts (10^5^ cells/ml) in the BALF. (e–h) Levels of IL-1*β*, IFN-*γ*, IL-4, and IL-17 in the BALF. (i, j) Representative hematoxylin and eosin (H&E) staining and inflammation score of lung sections of mice. (k, l) Representative PAS staining of lung sections and quantification of PAS-stained epithelial cells per bronchi showing airway mucus production in mice. (m–r) Flow cytometric analysis of percentages of TH2, TH17, and Treg lymphocytes in the lung.

OVA-sensitized mice developed features of allergic asthma, including increased airway hyperresponsiveness to methacholine (Figure 1(a)); increased bronchoalveolar lavage fluid (BALF) total cell and eosinophil counts (Figures 1(b) and 1(c)); higher BALF concentrations of IL-4, IL-1*β*, and INF-*γ* (Figures 1(e)–1(h)); perivascular and peribronchial leukocyte infiltration (Figures 1(i) and 1(j)); and increased bronchial mucus production (Figures 1(k) and 1(l)) when compared to control mice. OVA+LPS mice developed features of mixed eosinophilic and neutrophilic inflammation, including higher total cell and neutrophil counts (Figures 1(b) and 1(d)), higher Th17 but lower Treg percent (Figures 1(m)–1(r)), higher concentrations of BALF IL-17A (Figure 1(h)), severe airway hyperresponsiveness to methacholine (Figure 1(a)), more perivascular and peribronchial leukocyte infiltration (Figures 1(i) and 1(j)), and more bronchial mucus production (Figure 1(j)), but less eosinophil counts than OVA mice. There was no significant difference in BALF concentrations of IL-4, IL-1*β*, and INF-*γ* between OVA mice and OVA+LPS mice.

Simvastatin-treated OVA+LPS mice had reduced features of asthma as compared to PBS-treated counterparts, with reduced airway hyperresponsiveness (Figure 1(a)); lower BALF total cell and neutrophil counts (Figures 1(b) and 1(d)); reduced concentrations of BALF IL-4, IL-1*β*, INF-*γ*, and IL-17A (Figures 1(e)–1(h)); reduced perivascular and peribronchial leukocyte infiltration (Figures 1(i) and 1(j)); less mucus production (Figures 1(k) and 1(l)); and less lung Th2 and Th17, but higher percentage of Treg cells (Figures 1(m)–1(r)).

### 3.2. Simvastatin Treatment Ameliorates Severe Asthma through Reducing NETosis in Mouse Lungs

**Figure 2:**
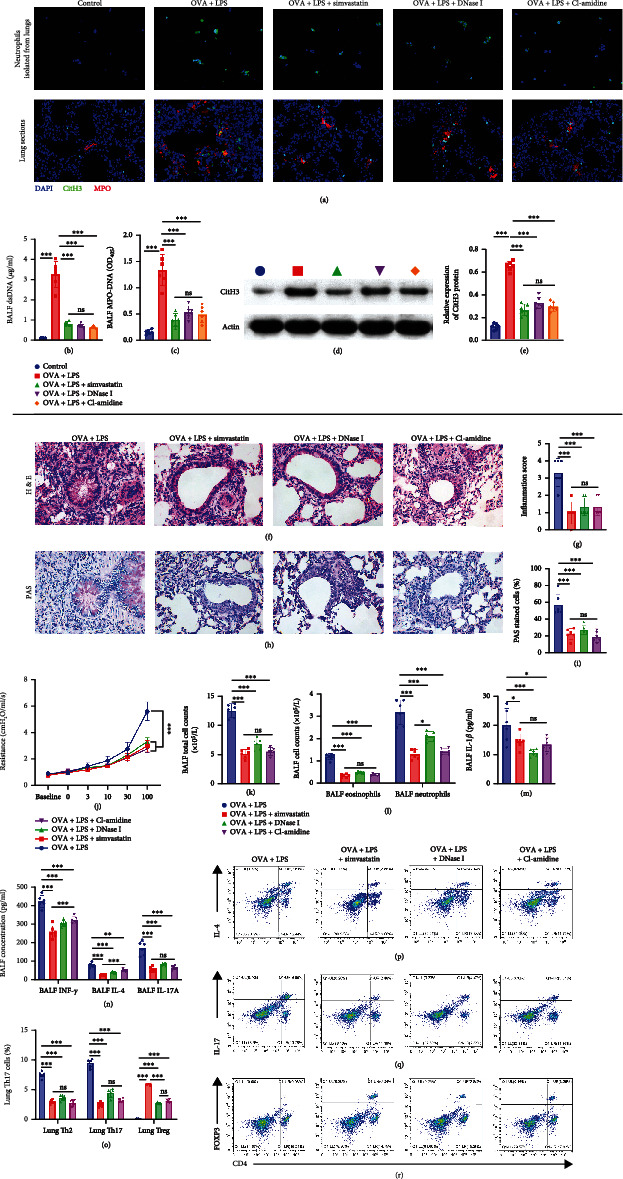
Simvastatin treatment attenuates TH17-mediated neutrophilic asthma through reducing NETosis in mouse lungs. (a) Confocal microscopy staining of CitH3^+^ MPO^+^ DAPI^+^ NETs released from ex vivo-cultured neutrophils and the lung sections of the indicated groups of mice. (b) Levels of extracellular dsDNA in the BALF. (c) ELISA measurement of MPO-DNA complexes in the BALF of mice. (d) Representative blots of CitH3 and actin (loading control) assessed by Western blot of lung protein extracts from mice. (e) Quantification of normalized CitH3 levels in lung protein extracts of mice. (f, g) Representative hematoxylin and eosin (H&E) staining and inflammation score of lung sections of mice. (h) Representative PAS staining of lung sections of mice. (i) Quantification of PAS-stained epithelial cells per bronchi showing airway mucus production in mice. (j) Measurement of dynamic airway resistance. (k, l) Total cell counts, eosinophil counts, and neutrophil counts (10^5^ cells/ml) in the BALF. (m, n) Levels of IL-1*β*, IFN-*γ*, IL-4, and IL-17A in the BALF. (o–r) Flow cytometric analysis of percentages of TH2, TH17, and Treg lymphocytes in the lung.

Lung neutrophils isolated from OVA+LPS mice released a great amount of NETs ex vivo, while lung neutrophils from control mice did not release any NETs ex vivo (Figure 2(a)). CitH3 in lungs and the amount of free double-stranded DNA (dsDNA) and of MPO-DNA complexes in BALF were higher in OVA+LPS mice than in the control mice (Figures 2(b)–2(e)). Furthermore, we observed that most CitH3 staining signals colocalize with MPO following OVA+LPS stimulation (Figure 2(b)).

DNase-I- or Cl-amidine-treated OVA+LPS mice showed a significant decrease of NET volume, including NETs in lung neutrophils isolated from OVA+LPS mice (Figure 2(a)), CitH3 in lungs (Figures 2(a), 2(d), and 2(e)), and the amount of dsDNA and of MPO-DNA complexes in BALF (Figures 2(b) and 2(c)). Furthermore, treatments with DNase I and Cl-amidine resulted in comparable reduction of asthma features to simvastatin-treated mice, including reduced perivascular and peribronchial leukocyte infiltration (Figures 2(f) and 2(g)); less mucus production (Figures 2(h) and 2(i)); reduced airway hyperresponsiveness (Figure 2(j)); lower BALF total cells, neutrophil counts, and eosinophil counts (Figures 2(k) and 2(l)); reduced concentrations of BALF IL-1*β*, INF-*γ*, IL-4, and IL-17A (Figures 2(m) and 2(n)); and lower lung Th2 and Th17 cell counts and higher Treg cell counts (Figures 2(o)–2(r)).

To investigate whether simvastatin treatment reduce NETosis, we examined the alteration of NET volume in simvastatin-treated mice. NET volume, including NETs in lung neutrophils isolated from OVA+LPS mice (Figure 2(a)), the amount of free double-stranded DNA (dsDNA) and MPO-DNA complexes in BALF (Figures 2(b) and 2(c)), and CitH3 in lungs (Figures 2(a), 2(d), and 2(e)) of simvastatin-treated mice, reduced significantly compared to vehicle-treated mice, but there were no significant differences compared to DNase-I- or Cl-amidine-treated mice.

**Figure 3:**
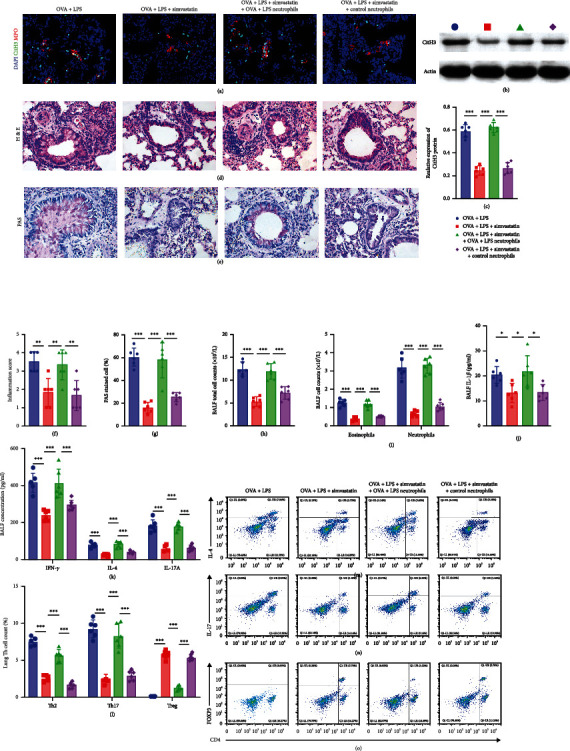
Adoptively transferring neutrophils from OVA+LPS mice to simvastatin-treated OVA+LPS mice was sufficient to trigger the TH17-mediated neutrophilic asthma. (a) Confocal microscopy staining of CitH3^+^ MPO^+^ DAPI^+^ NETs released from ex vivo-cultured neutrophils and the lung sections of the indicated groups of mice. (b) Representative blots of CitH3 and actin (loading control) assessed by Western blot of lung protein extracts from mice. (c) Quantification of normalized CitH3 levels in lung protein extracts of mice. (d, f) Representative hematoxylin and eosin (H&E) staining of lung sections of mice. (e) Representative PAS staining of lung sections of mice. (g) Quantification of PAS-stained epithelial cells per bronchi showing airway mucus production in mice. (h, i) Total cell counts, eosinophil counts, and neutrophil counts (10^5^ cells/ml) in the BALF. (j, k) Levels of IL-1*β*, IFN-*γ*, IL-4, and IL-17 in the BALF. (l–o) Flow cytometric analysis of percentages of TH2, TH17, and Treg lymphocytes in the lung.

To further investigate the role of NET formation during treatment of OVA+LPS mice with simvastatin, we adoptively transferred lung neutrophils from OVA+LPS mice to simvastatin-treated OVA+LPS mice. We found that the transferred neutrophils from OVA+LPS mice released a great amount of NETs (Figures 3(a)–3(c)), which was sufficient to trigger the asthmatic features including more perivascular and peribronchial leukocyte infiltration (Figures 3(d) and 3(f)); more mucus production (Figures 3(e) and 3(g)); higher total cell, neutrophil counts, and eosinophil counts (Figures 3(h) and 3(i)); higher concentrations of BALF IL-1*β*, INF-*γ*, and IL-4 and IL-17A (Figures 3(j) and 3(k)); and more lung Th2 and Th17 cell counts and less Treg cell counts (Figures 3(l)–3(o)).

### 3.3. Simvastatin Treatment Reduces NETosis via PAD4 in the Lungs of OVA+LPS Mice

**Figure 4:**
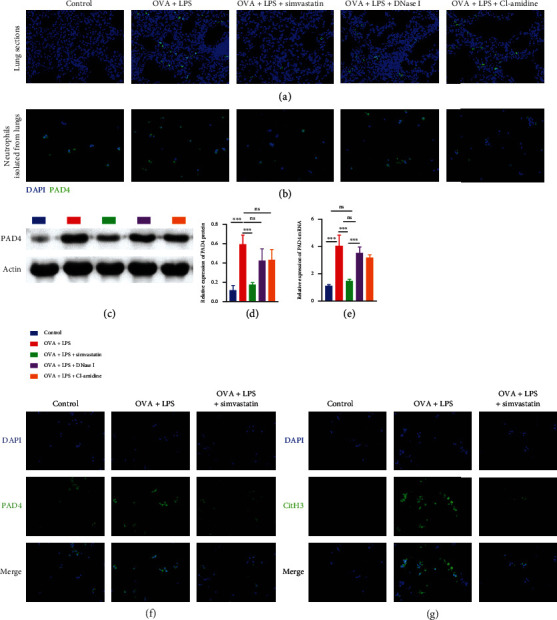
Simvastatin treatment reduces NETosis via PAD4 in the lungs of OVA+LPS mice. (a, b) Representative immunofluorescence images of PAD4 and DAPI staining of lung sections (*n* = 6) and ex vivo-cultured neutrophils in the indicated groups of mice. (c) Representative blots of PAD4 and actin (loading control) assessed by Western blot of lung protein extracts from mice. (d) Quantification of normalized PAD4 levels in lung protein extracts of mice. (e) The expression levels of PAD4 mRNA examined by RT-qPCR in lung homogenates of mice. (f, g) The lung neutrophils were isolated from OVA+LPS mice or control mice and preincubated the neutrophils in media with or without simvastatin (10 *μ*g/ml) before stimulation with 100 nmol/l phorbol 12-myristate 13-acetate (PMA). (f) Representative immunofluorescence images of PAD4 and DAPI staining. (g) Representative immunofluorescence images of CitH3 and DAPI staining.

To investigate the potential mechanisms underlying the impact of simvastatin on NET formation, we examined PAD4 expression in the lungs. Western blot and immunofluorescence analysis revealed a significant upregulation of PAD4 protein expression in the lungs of OVA+LPS mice and the neutrophils isolated from the lungs as compared to the control mice (Figures 4(a)–4(d)). Simvastatin treatment significantly reduced PAD4 protein expression in OVA+LPS mice, while DNase-I or Cl-amidine treatment did not affect PAD4 protein expression (Figures 4(a)–4(d)). PAD4 mRNA expression showed similar increase in lungs of OVA+LPS mice and reduction by simvastatin treatment as PAD4 protein, when determined by RT-PCR (Figure 4(e)).

Next, we isolated neutrophils from the lungs of OVA+LPS and control mice and preincubated the neutrophils in media with or without simvastatin (10 *μ*g/ml) before stimulation with 100 nmol/l phorbol 12-myristate 13-acetate (PMA). We found that neutrophils isolated from lungs of OVA+LPS mice had a significant increase in PAD4 expression and NET formation (Figures 4(f) and 4(g)) upon stimulation with PMA compared to control mice and that the stimulated neutrophils of OVA+LPS mice had a significant reduction in PAD4 expression and NET formation when preincubated with simvastatin (Figures 4(f) and 4(g)).

### 3.4. Simvastatin Treatment Reduces NET Formation in Neutrophils via PAD4 In Vitro

To further confirm the role of PAD4 in decrease of NET formation during simvastatin treatment, we examined the expression of PAD4 and formation of NETs in differentiated HL-60 neutrophil-like cells in vitro.

**Figure 5:**
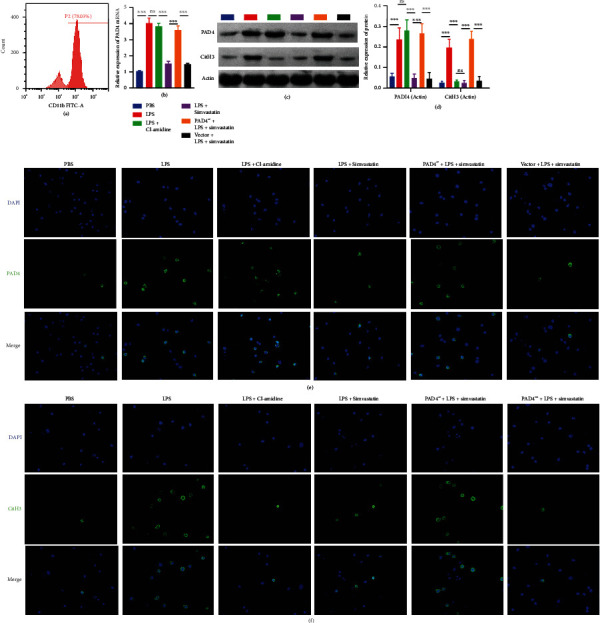
Simvastatin treatment reduces NET formation in neutrophils via PAD4 in vitro. (a) Flow cytometry analysis of CD11b expression on HL-60 cells after 5 days of culture with DMSO. (b) The expression levels of PAD4 mRNA examined by RT-qPCR in the HL-60-differentiated neutrophil-like cells. (c) Representative blots of PAD4 and actin (loading control) assessed by Western blot of the HL-60-differentiated neutrophil-like cells. (d) Quantification of normalized PAD4 levels in the HL-60-differentiated neutrophil-like cells. (e) Representative immunofluorescence images of PAD4 and DAPI staining in the HL-60-differentiated neutrophil-like cells. (f) Representative immunofluorescence images of CitH3 and DAPI staining in the HL-60-differentiated neutrophil-like cells.

Flow cytometry analysis of CD11b expression on HL-60 cells revealed that about 80% of HL-60 cells were differentiated to neutrophil-like cells after cultured in the presence of 1.25% DMSO for 5 d (Figure 5(a)). LPS treatment significantly increased PAD4 mRNA (Figure 5(b)) and protein expression and NET formation in the differentiated neutrophil-like cells compared to vehicle treatment upon stimulation with PMA (Figures 5(c)–5(f)). Preincubation with Cl-amidine or simvastatin significantly reduced NET formation in LPS-treated neutrophil-like cells, but simvastatin reduced PAD4 expression significantly while Cl-amidine did not (Figures 5(b)–5(f)). Furthermore, PAD4-overexpressed lentivirus transduction restored PAD4 expression and NETosis in simvastatin-treated HL-60-differentiated neutrophil-like cells (Figure 5(b)–5(f)).

## 4. Discussion

Here, we established a mouse model of severe asthma with neutrophil predominant inflammation. NET volume in lungs increased significantly in OVA+LPS mice. Simvastatin treatment reduced features of nontype 2 neutrophilic inflammation and airway hyperresponsiveness in OVA+LPS mice. Simvastatin treatment reduced NETosis significantly by inhibition of PAD4 expression in lungs of OVA+LPS mice and HL-60-differentiated neutrophil-like cells.

Asthma is a heterogeneous disorder. Type 2 inflammation is prominent in more than half of asthmatic patients [25], but only in 37% of severe asthmatic patients [26]. Severe asthmatic patients respond poorly to corticosteroids and have substantial neutrophilia that may indicate distinct pathogenic mechanisms (endotypes). Previous animal studies using systemic statins for the treatment of allergic asthma demonstrated decreased airway hyperresponsiveness, reduced BALF cell count, cytokines, and reduced airway mucus production [27–30]. An in vitro asthma study showed that the combination of a statin and a corticosteroid could enhance anti-inflammatory effect by augmenting the Treg/Th17 cell ratio in asthma patients [31]. So that statins may be more effective in severe asthmatic patients with nontype 2 airway inflammation rather than mild or moderate asthmatic patients [31, 32]. But to our knowledge, there was no animal study concerned with statin use in nontype 2 neutrophilic asthma. In our OVA+LPS mouse models, simvastatin treatment ameliorates not only conventional asthmatic features but also neutrophilic inflammation features including reduced BALF neutrophil counts, lower BALF concentration of IL-17A, and less lung Th17 but more lung Treg counts. These results indicate that simvastatin treatment is effective for Th17-mediated neutrophil predominant asthmatic mice. Furthermore, a retrospective, cross-sectional study of 165 patients with severe asthma at a single center showed that statin users had improved asthma symptom control, reduced risk of acute exacerbations, and need for rescue bronchodilator use compared to nonusers [33]. Our study provides in vivo evidence that simvastatin augmented Treg/Th17 cell ratio in response to airway inflammation and thus may be more effective in Th17-mediated neutrophil predominant asthma.

It was found that severe asthmatic patients had higher circulating, BALF, and sputum NET levels [5, 34–36], and NET level was an independent predictor of poor control [34]. NETs can mediate the onset and exacerbation of allergic airway inflammation in asthmatic mouse models [6, 7, 37]. Furthermore, IL-17A production is downstream of NETosis for lung neutrophil recruitment [5].

In this study, we found that NET formation increased dramatically in LPS+OVA mice. We used DNase I, which degrades NETs, and Cl-amidine, which inhibits NETosis, to assess the functional importance of NETs in the initiation of Th17-mediated neutrophilic airway inflammation in this severe asthmatic model. We found that treatment with DNase I and Cl-amidine reduced neutrophil and Th17 counts and IL-17A production in lungs of OVA+LPS mice and protected the mice on the development of asthma, suggesting that NETs could mediate the Th17-mediated neutrophilic airway inflammation in the OVA+LPS mice models tested here.

Furthermore, simvastatin treatment showed similar decrease of NETosis and had the same global outcome as Cl-amidine and DNase I on the development of severe asthma, implicating that inhibition of asthmatic airway inflammation by simvastatin may be mediated by decrease of NETosis. Next, we adoptively transferred neutrophils from OVA+LPS mice to simvastatin-treated OVA+LPS mice and found that the transferred neutrophils were sufficient to restore NETosis and trigger Th17-mediated neutrophilic airway inflammation in OVA+LPS mice. Together, these findings suggest that simvastatin treatment attenuates severe asthma in mouse models at least partly through inhibition of NETosis.

NETosis releases NETs from neutrophils, which may result in the formation of enucleated cell bodies called cytoplasts [5]. Krishnamoorthy et al. reported neutrophil cytoplast formation in asthmatic lung inflammation and linked the cytoplasts, but not NETs, to Th17-mediated neutrophilic inflammation in severe asthma [5], whereas two other studies showed that LPS- and rhinovirus-induced NETs contribute to allergic airway inflammations, and the airway inflammations can be protected by degrading NETs with DNase [6, 7]. In this study, IL-17A reduced significantly in OVA+LPS mice treated with DNase I, which implicate that, to a certain extent, the NETs are likely to induce the Th17-mediated neutrophilic inflammation. But our data do not exclude the potential role of cytoplasts during the treatment of Th17-mediated neutrophilic inflammation by simvastatin and Cl-amidine, since cytoplasts are enucleated cell bodies derived from NETosis, which can be inhibited by simvastatin or Cl-amidine.

Studies have shown that NETs were exclusively derived from neutrophils in mice with rhinovirus-induced asthma exacerbations [6] and allergic asthma mice exposed to allergen with LPS [5, 7]. However, a recent study by Lu et al. showed that eosinophil extracellular traps were associated with OVA-sensitized allergic asthma progression [38]. In OVA+LPS mice of this study, we observed most NETs colocalizing with MPO in lungs by immunofluorescence and increased MPO-DNA complexes in BALF. These findings implicate that the NETs in lung of our OVA+LPS mice were mainly derived from neutrophils.

Several clinical studies showed that NET volumes in circulation, sputum, or BALF appeared to be good markers of asthma severity, poorer control, and more frequent oral steroid use [5, 34–36]. These studies suggest that targeting NETosis may be a novel treatment for severe asthmatic patients with high levels of circulating or BALF NETs [35, 36, 39] In this study, MPO-DNA and dsDNA increased significantly in BALF of OVA+LPS mice, and simvastatin significantly reduced MPO-DNA and dsDNA in BALF of OVA+LPS mice. Given the crucial role of NETosis in progression of severe asthma, we speculated that high BALF and/or NET volume in blood might be biomarkers of a subset of severe asthmatic patients who may respond well to simvastatin treatment. Further clinical studies are needed to determine whether the subpopulation of severe asthma with high BALF or circulating NET volume can be a reasonable candidate for statins treatment.

Studies have revealed the NETosis inhibiting effect of simvastatin in patients with sepsis [19] or a mouse model for thermal injury [20], but the exact mechanisms are still unknown. PAD4 is the essential enzyme during NET formation [40, 41]. Studies showed that inhibition of PAD4 activity blocks NET formation in mouse models of asthma, sepsis, and cancer [7, 42, 43]. Moreover, LPS is sufficient to induce neutrophilic PAD4 expression and subsequent NETosis [44], and PAD4-deficient mouse neutrophils fail to release NETs upon LPS stimulation [45]. In asthmatic mouse models, during HDM/LPS sensitization, PAD4 KO or Cl-amidine-treated mice had reduced NET formation and airway inflammation in lungs [5, 7]. Hence, we determined whether decrease of NETosis by simvastatin is mediated by inhibition of PAD4 activity. We found that mRNA and protein levels of PAD4 were significantly upregulated in lungs of OVA+LPS mice and in neutrophils isolated from lungs of OVA+LPS mice. Simvastatin treatment significantly reduced the upregulated mRNA and protein levels of PAD4 and subsequent NETosis. These results indicate that the upregulation of PAD4 expression in neutrophils contributes to the increase of NETosis in lungs of OVA+LPS mice and that simvastatin treatment can reduce both mRNA and protein levels of PAD4. In line with this hypothesis, we also found that simvastatin significantly reduced LPS-induced upregulation of PAD4 expression and NETosis in HL-60-differentiated neutrophil-like cells, and PAD4-overexpressed lentivirus transduction restored PAD4 expression and NETosis in simvastatin-treated HL-60-differentiated neutrophil-like cells. These results demonstrated that simvastatin reduced NETosis through downregulation of PAD4 expression.

The pathways that regulate the PAD4 activity and expression are incompletely understood. Activation of PAD4 by hydrogen peroxide requires PKC*ζ* or calcium regulated by PI3K [40]. Wolach et al. find that PAD4 is upregulated in JAK2-mutated neutrophils and hypothesize that JAK2-STAT may activate the PAD4 expression [46]. A study by Wong et al. demonstrates that diabetes can activate neutrophils to upregulate PAD4 expression, but the exact mechanism is still uncovered [47]. Most recently, Ou et al. find that TcpC can serve as an E3 ligase that promotes degradation of PAD4 [44]. In our study, we demonstrate that simvastatin inhibits the transcription of PAD4, but the exact mechanism still needs to be uncovered.

In summary, this study demonstrates that simvastatin reduces Th17-mediated neutrophilic airway inflammation and airway hyperreactivity by inhibition of NETosis in OVA+LPS mice, and the inhibitory effect of simvastatin on NETosis is primarily mediated by reducing transcription of PAD4. Our findings provide not only a novel mechanism by which simvastatin ameliorates the Th17-mediated neutrophilic airway inflammation but also new clues to mark a subset of severe asthmatic patients with high circulating or sputum NETs who may respond to statin treatment well.

## Data Availability

The authors confirm that all data underlying the findings are available. All relevant data are within the paper.
